# What Are the Acute and Chronic Effects of Initial Military Training on Physiological and Neuromuscular Performance in Military Populations? A Narrative Review

**DOI:** 10.1007/s40279-026-02431-6

**Published:** 2026-04-13

**Authors:** Sean C. McCleary, Aaron Uthoff, Matt R. Cross

**Affiliations:** Sports Performance Research Institute New Zealand (SPRINZ) at AUT Millennium, Auckland University of Technology, Auckland, New Zealand

## Abstract

Initial military training (IMT) is designed to physically and mentally transition recruits from civilian to military personnel, typically lasting 6–14 weeks. Accordingly, the content and focus of IMT appear variable, and it is important to understand what effects this training has on recruits. This study reviewed existing literature regarding the acute and chronic effects of IMT on physiological and neuromuscular performance. Using a systematic style search strategy (Google Scholar, EBSCO, PubMed, Medline), 28 relevant studies were identified, focusing on longitudinal (*n* = 26) and acute (*n* = 6) effects. The included studies were performed in various branches of the military, but primarily the army, across nations. They rarely disclosed the nature of activities involved, but tended to follow a progressive structure, culminating in scenario-based training specific to their respective branch, to emphasise skills learned earlier in the course. Findings indicate that training causes an acute decrease in strength and power, along with increased cardiovascular strain and maximal oxygen consumption, with these effects being more pronounced in females. Longitudinally, recruits demonstrated improvements in cardiorespiratory endurance, full-body strength and upper-body muscular endurance; however, performance gains typically plateaued by the eighth week. This plateau, most evident in recruits with higher baseline fitness, suggests potential fatigue accumulation from sleep restriction and high physical activity, or a late-stage shift in training focus that provides insufficient stimulus. These results underscore a critical need for standardised assessment protocols to address literature heterogeneity and enhance the comparability of training outcomes across military populations.

## Key Points

**Table:** 

Initial military training organisational practices vary widely in duration, intensity and design. In addition, inconsistent outcome measures complicate interpretation; for example, changes may reflect true gains in maximal capacity or improvements driven by test-specific thresholds (e.g. clustering around minimum passing standards).	
Broadly, neuromuscular strength and power appear to decrease immediately following activities core to initial military training (i.e. load carriage). Cardiovascular strain and *V*O_2_ consumption increase significantly during physical activity and are more prevalent in females.	
IMT training appears to improve cardiorespiratory endurance, full body strength, upper-body muscular endurance and power. Improvements primarily occur during the first eight weeks, particularly in females and in individuals with lower initial fitness levels.	
Future research should prioritise the establishment of standardised physical assessment protocols across military cohorts. Large-scale, longitudinal studies with similar data collection methods are essential to improve comparability and to develop a more cohesive understanding of how specific training modalities influence long-term health and readiness.	

## Introduction

Life in the military (e.g. Army, Air Force and Navy) is demanding. The role of military personnel varies by nation, branch of the military, specific role and level of rank (among other factors), but all must be prepared for a range of tasks, including wartime deployments, peacekeeping and disaster relief [1]. Tasks often involve strenuous physical activity, including combat engagement, marching, digging and marksmanship, which at times are performed under high cognitive and physical demands (e.g. sleep deprivation). A critical part of these tasks is movement, which often occurs while carrying significant loads (e.g. a pack with food, water, ammunition and other survival equipment) and operating in stressful environments. In particular, heavily loaded marching is commonplace and taxing; for example, soldiers deployed in Afghanistan carried a mean load of 57 kg [2]. As personnel must be mentally and physically fit for service, the military strives to maintain strict physical fitness standards. Initial military training (IMT), often referred to as recruit training, boot camp or basic training, is a mandatory high-intensity course typically lasting 6–14 weeks [3]. It is designed to turn civilians into military personnel, serving to improve occupation-specific skills, physical readiness and discipline. While the training regimen and demands of these courses may differ significantly, they are all designed to physically and mentally challenge recruits, with sleep deprivation, caloric deficits, load carriage and psychological stress to prepare them for potentially extreme situations.

Research shows that improved cardiorespiratory fitness reduces the physical strain of military personnel and enhances recovery between activities [4]. Furthermore, higher levels of fitness are also closely linked to subsequent performance in specific operational tasks [5–8] and help to reduce injury risk [9–11]. It is well documented that initial military training increases cardiorespiratory fitness [3], but also features a high prevalence of injuries [12, 13], which often result in high levels of attrition and financial burden [14]. This is in part due to the predominantly high endurance-based load of training [15, 16], exposing recruits to high physical stressors with little to no preparation. This is heightened by the modern recruit’s increasingly sedentary lifestyle before entry [17–19]. Recruits also face significant cognitive load in addition to physical training stressors [15]. Furthermore, during IMT, there is often inadequate energy consumption [20] or time allowed for recovery, with research identifying that up to 85% of recruits are underslept [21], which is of concern as Belenky et al. [22] have noted that 8 h of sleep is optimal, with just 1 h less causing declines in cognitive performance. Giles et al. [23] found that higher loads were most strongly associated with declines in cognitive function and increased perceived exertion [40], aligning with recent systematic research indicating that the effect was greater in female recruits [24, 25], who are at a significantly greater risk of injury than males [26, 27]. Accordingly, an understanding of why these sex-based differences might occur is critical for maintaining an effective military force by mitigating potential risk. Combined, these factors create a perfect storm for fatigue, which is a performance loss from poor sleep, stress or exertion and can be split into central (neural drive) and peripheral (motor control) types [28, 29]. Although distinct, they often overlap: central issues like sleep loss impair physical performance [28], while peripheral exertion rarely causes central fatigue. This complexity makes them hard to isolate, yet both consistently degrade vital reaction times, marksmanship accuracy, strength and vertical jump performance [30, 31]. While the effects of fatigue may be deliberately imposed at times, there is a clear lack of individualised periodisation or intervention targeting the effects of fatigue and the subsequent predisposition to injury that recruits may face if it is left unmanaged [21].

The training that occurs during IMT differs between branches of the military and between nations, making it difficult to fully understand the effects and outcomes of the training. However, there is a lack of breadth in the literature; as such, this review synthesises the available literature to help form a greater understanding of the effects of IMT more broadly. Generally, IMT follows a progressive structure where recruits are slowly exposed to increasing stressors, such as physical tasks including load carriage, manual material handling and casualty evacuation [8]. Load carriage, in particular, is frequent and among the most physically demanding tasks conducted by recruits [8], requiring high levels of physical fitness. They develop their physical fitness through a combination of physical training, corrective exercises and scenario-based training. This training often focuses on endurance-based qualities, despite recent literature suggesting that concurrent strength and aerobic training is optimal to enhance performance during physically demanding tasks such as load carriage [4, 8, 32, 33]. In addition, development of some qualities may be impacted by an interference effect, which can inhibit the development of neuromuscular strength and power when not carefully periodised [34]. They also participate in classroom sessions to learn essential skills relevant to their branch of the military, which may include live firing, tactics and drills. Often, these aspects are combined, where skill-based qualities will be tested under physical and/or mental duress, such as marksmanship during or following tactical manoeuvres. This approach trains recruits to operate in a wartime scenario where they are likely to face fatigue and high stress. Ideally, recruits should enter with a base level of neuromuscular and physiological fitness to ensure they can withstand its demands. This is often assessed before and/or early in the course and is further developed as it continues. The physical assessments tend to mimic those used throughout all levels of the respective branch, such as runs for time, push-ups and sit-ups [35, 36]. Some branches have begun expanding and improving their testing methods, such as maximum strength and vertical jump testing [32, 35, 37–42], with the aim of improving insight into the adaptations that occur. Unfortunately, a complicating factor in synthesising results drawn from official military assessment events is whether recruits rate-limit their performance to meet the passing threshold, or whether they represent true maximal intent. This makes it difficult to draw concise conclusions. Accordingly, while these tests often have different age groups or sex standards [43], operational scenarios are non-discriminatory; all personnel must carry the same loads and engage in the same combat scenarios.

Despite the rigorous nature of initial military training, a significant gap remains in our understanding of how these elevated physical and cognitive loads influence recruit performance and injury risk. While traditional assessments provide a snapshot of fitness, they often fail to capture the nuanced physiological and neuromuscular fatigue that predisposes recruits, particularly females [26], to injury and attrition. As demands remain uncompromising regardless of a recruit's background, there is a need to synthesise the current literature on these stressors. Given the scarcity of recruit literature, a broader lens was taken to encompass all military branches. Furthermore, training regimens and methodology are not fully transparent in the literature, making it difficult to draw valid conclusions. Therefore, this review aims to collect and synthesise the existing literature specifically relating to the acute and chronic effects of IMT on physiological and neuromuscular performance in military populations, with a specific focus on the sex-based differences. By doing so, it seeks to provide a foundation for more individualised training and periodisation, by ultimately enhancing responses to training during IMT.

## Methods

### Literature Search Strategy

This review followed a narrative format with a systematic search strategy. A predetermined search string was validated by all authors, utilising combinations of key terms: (“military”, “army”, “navy”, “air force”) AND (“basic training” OR “basic military training” OR “initial military training”) AND (“neuromuscular”, “fatigue”, “performance”, “strength”, “power”, “cardiovascular”, “cardiorespiratory”, “loaded marching” OR “load carriage”). The search strategy was set to encompass all military branches to strengthen the breadth of literature included in the review. Selection bias was further mitigated through the application of stringent inclusion and exclusion criteria. Data were extracted using a standardised approach, ensuring that all relevant findings were synthesised regardless of the direction of the results, thereby providing a balanced overview of the current evidence base.

Per the aims of the review, we set out to capture literature pertaining to the long (chronic) and short-term (acute) effects of IMT more broadly, and load carriage more specifically (as a critical element of IMT). The long-term effects were categorised by pre- and post-measurements taken at the start and end of the course, with studies presented first that did not feature any form of intervention (i.e. the effects of an unaltered course). Short-term effects were assessed immediately following an activity but no later than 3 days afterwards. Furthermore, elements of physical fitness were categorised into four areas: cardiorespiratory fitness, maximal strength, muscular endurance and muscular power. Cardiorespiratory fitness was defined as the combined ability of the heart and lungs to provide oxygen to the muscles during sustained activity. This was measured through maximal oxygen uptake, validated aerobic tests or any activity relying primarily on the aerobic energy system. Maximal strength was identified as either a short-duration maximal isometric or the heaviest load lifted during dynamic strength assessments (e.g. one-repetition maximum). Muscular endurance was defined as sustained submaximal strength assessments. Muscular power was defined as the capacity to produce maximal work per unit of time, quantified through assessments measuring the product of force and velocity.

### Inclusion Criteria

The inclusion criteria required that articles identified through the search strategy: (1) be peer reviewed, (2) be written in English or have a relevant translation available, (3) involve a population of military recruits, (4) be related specifically to the effects of IMT, (5) include participants that were injury free, (6) use an acute and/or longitudinal study design and (7) contain methods capable of objectively quantifying relevant variables. All articles that did not meet the above criteria were excluded. The study selection process involved removing duplicates, screening titles and abstracts for relevance and, finally, screening the full-text articles and their reference sections for additional relevant literature, using the inclusion criteria.

## Results

### Study Characteristics

**Fig. 1:**
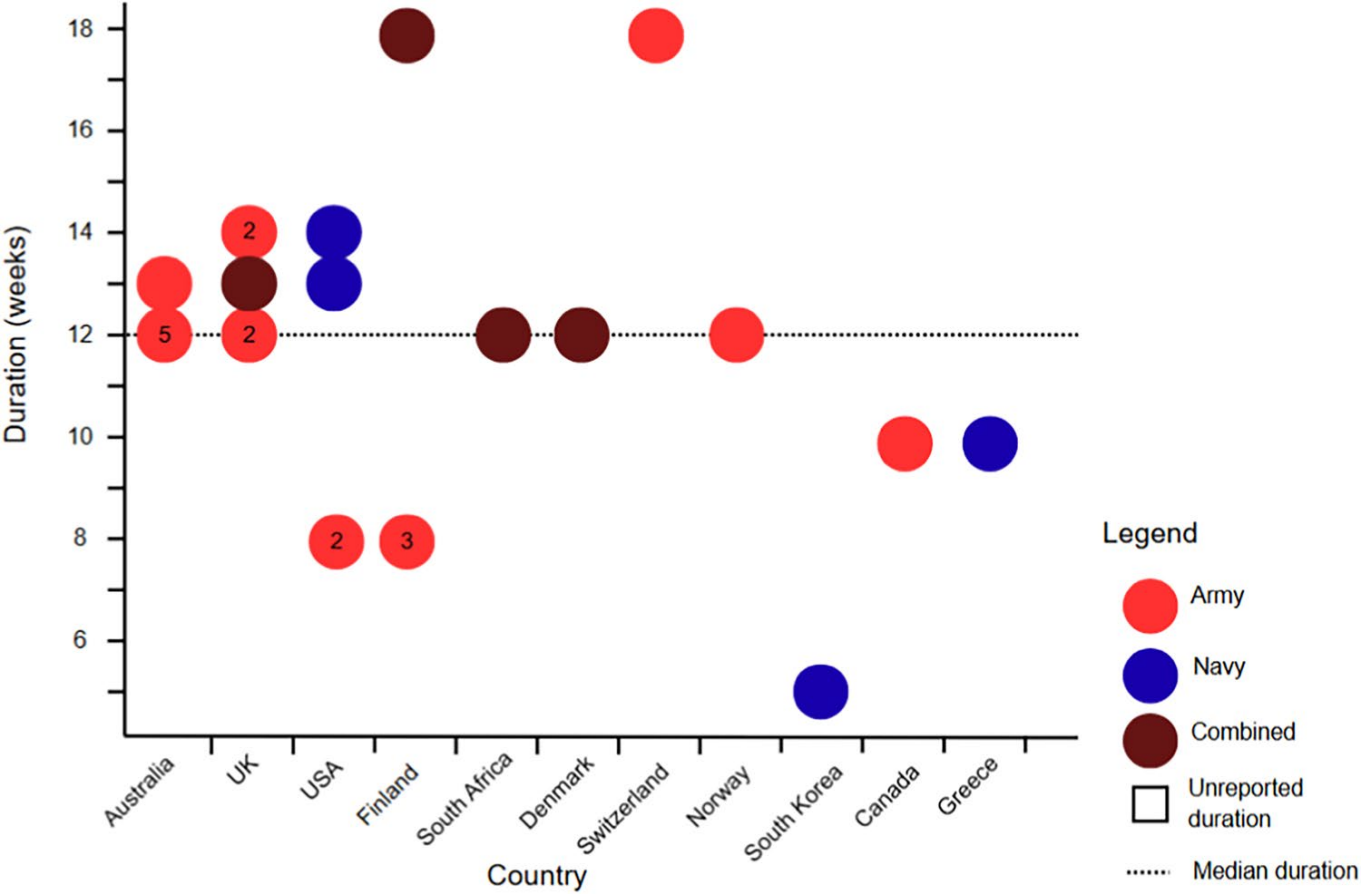
Reported basic training durations by country, military branch and study weight. Note: numbers correspond to *N* studies

Through the literature search, 28 studies were identified that examined the acute and chronic effects of IMT. Six studies featured investigations into the acute effects of IMT and were included after the inclusion criteria were applied. These studies totalled 1244 recruits (male, 822; female, 422) from Finland, Norway, UK and USA, and spanned 5–18 weeks in duration. Twenty-six longitudinal studies investigating chronic effects were identified, totalling 6184 recruits (male, 4442; female, 1142; unspecified, 600) from Australia, Canada, Denmark, Finland, Greece, Norway, South Africa, South Korea, Switzerland, UK and USA. Of the 28 studies included, 26 focused more broadly on the overall effects of IMT, while 3 focused on the effects of load carriage. All literature included is presented in Table 1. In addition, studies in non-recruit populations used to bolster sections lacking sufficient data are presented in Table 2. For the included chronic studies, Fig. 1 presents the durations, country and branch of military, including the frequency with which the specified duration appeared in the included studies.

**Table 1:** Studies included in literature search (*n* = 28)

Study	Subjects (sex, mean age ± SD, weight ± SD)	Branch	BT Duration	Test	Outcome
Santtila et al. (2012) [34]	57 males, 19.2 ± 0.9 years, 73.8 ± 12.4 kg	Finnish Military	16 weeks	Cardiorespiratory endurance Lower-body strength Upper-body strength	↑ *V*O_2_ peak 5.6% (*p* < 0.001) ↑ Isometric leg extension 3.8% (*p* < 0.001) in first 8 weeks, no further change ↑ Isometric arm extension 8.1% (*p* < 0.001) in first 8 weeks, no further change
Santtila et al. (2008) [40]	72 males, 19.4 ± 0.8 years, 69.7 ± 8.5 kg	Finnish Army	8 weeks	Cardiorespiratory endurance Lower-body strength	↑ *V*O_2_ peak NT: 13.4% (*p* < 0.001), ST: 12% (*p* < 0.01), ET: 8.5% (*p* < 0.05) ↑ Isometric leg extension NT: 5.2% (*p* = 0.45), ST: 9.1% (*p* < 0.05), ET: 12.9% (*p* < 0.01)
Rue et al. (2023) [41]	339 males, 42 females, JE: 16 ± 1 years, SE: 21 ± 4 years, RAF: 21 ± 3 years, JE: 68.6 ± 9.2 kg, SE: 71.7 ± 11.1 kg, RAF: 71.4 ± 9.9 kg	British Army and Air Force	13 weeks	Cardiorespiratory endurance Lower-body strength Upper-body power	2 km run time ↓ for all (JE:2.1%, *p* < 0.001, SE: 4.8%, *p* < 0.001, RAF: 4.5%, *p* < 0.001) IMTP ↑ for JE 10.8% (*p* < 0.001), SE and RAF no change Medicine ball throw (4 kg) ↑ for JE 6.8% (*p* < 0.001), SE 2.3% (*p* = 0.04), ↓ for RAF 1.4% (*p* = 0.02)
Larsen et al. (2022) [21]	57 males, 11 females, 23 ± 5 years, 76 ± 15 kg	Australian Army	12 weeks	Sleep duration and efficiency	Mean 06.24 ± 00.18 h per night ↓ Sleep time (06:06 ± 00:36 h) and efficiency in field (*p* < 0.01)
Wood and Kruger (2013) [36]	173 males, 200 females, NT: male 20.5 ± 3.4 years, female = 19.9 ± 3.1 years, EXP: male: 20.2 ± 3.3 years, female: 20.0 ± 3.2 years, NT: male 62.3 ± 6.7 kg, female: 59.1 ± 8.7 kg, EXP: male: 61.8 ± 6.9 kg, female: 60.2 ± 9 kg	South African Military	12 weeks	Cardiorespiratory endurance Upper-body endurance Lower-body endurance	2.4 km run time NT ↑ 6% (*p* = 0.001), EXP ↓ 12% (*p* = 0.0001) ↓ Shuttle run test (22 m × 10) time 6% (*p* = 0.0001) ↓ 4 km walk time 6% (*p* = 0.0001) ↑ Push-up NT: Improve 37%, EXP: 91%, (*p* < 0.05) ↑ Sit-up NT: 62%, EXP: 90%, (*p* < 0.05)
Santtila et al. (2010)^b^ [32]	72 males, 19.2 ± 0.9 years, 73.8 ± 12.4 kg	Finnish Army	8 weeks	Cardiorespiratory endurance Lower-body strength	↑ *V*O_2_ peak NT: 13.4% (*p* < 0.001), ST: 12.0% (*p* < 0.01), ET: 8.5% (*p* < 0.05) ↓ 3 km loaded march time NT: 10.2%, ST: 12.4%, ET: 11.6% (*p* < 0.001) ↓ Isometric leg extension (following loaded march) 10% (NT:* p* < 0.001, ST and ET *p* < 0.05)
Harman et al. (2008) [35]	32 males, ST: 27 ± 4.7, NT: 29 ± 4.6 years, ST: 80.9 ± 12.7 kg, NT: 84.5 ± 10.4 kg	Civilian (following a US Army BT programme)	8 weeks	Cardiorespiratory endurance Upper-body strength Lower-body strength Upper-body endurance Lower-body power	↓ 3.2 km loaded run (32 kg) time ST: 15%, NT: 14% (*p* < 0.05) ↓ 400 m loaded run (18 kg) time ST: 16%, NT: 11% (*p* < 0.05) ↓ Obstacle course time ST: 10%, NT: 16% (*p* < 0.05) ↓ 30 m rush × 5 time ST: 2%, NT: 6% (*p* < 0.05) ↓ Casualty rescue time ST: 23%, NT: 36% (*p* < 0.05) ↑ *V*O_2_ peak ST:13%, NT: 10% (*p* < 0.05) ↑ 1RM bench press ST: 12%, NT: 11% (*p* < 0.05) ↑ 1RM back squat ST: 12%, NT: 10% (*p* < 0.05) ↑ Push-up ST: 32%, NT: 31% (*p* < 0.05) ↑ Sit-up ST: 28%, NT: 50% (*p* < 0.05) ↑ Vertical jump ST: 5%, NT: 1% (*p* < 0.05) ↑ Horizontal jump ST: 3%, NT: 3% (*p* < 0.05)
McFadden et al. (2024) [42]	182 males, 99 females, 19 ± 2 years, male: 73.9 ± 10.4 kg, female: 61.7 ± 8.1 kg	US Marines (Navy)	13 weeks	Lower-body strength Lower-body power Sleep duration	IMTP relative peak force, no change IMTP peak force, no change ↓ CMJ relative peak power (male: 11%, female: 5% (*p* < 0.001) ↓ CMJ peak power (male: 10%, female: 5% (*p* < 0.001) Mean time 6.2 ± 1.1 h per night
Wyss et al. (2014) [48]	600 (sex unspecified), 20.7 ± 1.2 years, 73.7 ± 10.6 kg	Swiss Army	18 weeks	Sleep duration	Mean time 6.5 h per night
Rosendal et al. (2003) [39]	330 males, 20 ± 1 years, well trained: 77.2 ± 0.8 kg, trained: 79.2 ± 0.7 kg, less trained: 79.4 ± 1.2 kg, untrained: 85.9 ± 2.5 kg	Danish Military	12 weeks	Cardiorespiratory endurance Lower-body power	Multistage fitness test, untrained: ↑ 62%, all others no change Coopers 12-min run test, untrained: 16%, trained 4%, others no change CMJ (unloaded), ↓ well trained: 13%, trained: 9%, less trained: 12% (*p* < 0.05), untrained: no change CMJ (15-kg pack), ↓ well trained, trained, less trained (*p* < 0.05), untrained: ↑ 9% (*p* < 0.05)
Saner et al. (2024) [53]	1318 males, 263 females, 22.4 ± 5.1 years, 75.5 ± 17.1 kg	Australian Army	12 weeks	Cardiorespiratory endurance	↑ Multistage fitness test 6.6% (*p* < 0.001, ES 0.58), male: 10.8 ± 7.9%, female: 3.1 ± 6.8%
Ahn et al. (2020) [44]	270 males, 15 females, male: 18.83 ± 0.76, female: 18.18 ± 0.54 years, weight unspecified	Korean Navy	5 weeks	Cardiorespiratory endurance Upper-body endurance Mood profile	↓ 3-km run time 17% (NS) ↑ Push-up 45% (NS) ↑ Sit-up 21% (NS) Weeks 1–5 spiked anger and fatigue (NS). Initial fitness had no relationship with mood or fatigue
Burley et al. (2018) [37]	173 males, 22 females, 21.5 ± 4.2 years, 76.2 ± 12.4 kg	Australian Army	12 weeks	Cardiorespiratory endurance Lower-body strength Upper-body endurance	↓ 3.2-km loaded carry (22 kg) 9% (*p* < 0.05) ↑ Multistage fitness test 9% (*p* < 0.001) ↑ 1RM box lift (1.5 m) 22% (*p* < 0.001) ↑ Push up 11% (*p* = 0.018)
Bulmer et al. (2022a) [45]	29 males, 6 females, 24.8 ± 6.8 years, 75.6 + 14.7 kg	Australian Army	12 weeks	Cardiorespiratory endurance Wellness questionnaire	↑ Multistage fitness test (*p* < 0.001) ↑ Reported stressors in field (NS)
Bulmer et al. (2022b) [47]	37 males, 8 females, 25.2 ± 7.2 years, 76.8 ± 15 kg	Australian Army	12 weeks	Sleep duration Sleep efficiency Sleep questionnaire	Mean time 6.3 ± 1.2 h per night Efficiency 85.6% ± 5.5% Sleep quality had a strong relationship with stress, fatigue. Sleep was worse at the start of BT
Vantarakis et al. (2022) [52]	153 males, 32 females, 18.3 ± 0.6 years, 75.3 ± 7.6 kg	Greek Navy	10 weeks	Cardiorespiratory endurance	↑ Coopers 12-min run test 10.8% (*p* < 0.01)
Burley et al. (2020) [38]	162 males, 52 females, 21.7 ± 3.9 years, 73.4 ± 12.4 kg	Australian Army	12 weeks	Cardiorespiratory endurance Upper-body strength Lower-body strength Upper-body endurance Lower-body power	↓ 3.2 km load carriage (22 kg) time, EXP: 11.9%, NT: 8% (*p* = 0.030, ES: -0.30) ↑ Multistage fitness test, EXP: 13.2%, NT: 8% (*p* = 0.022, ES: 0.58) ↑ 1RM bench press, EXP: 26.3, NT: 17% (*p* < 0.001, ES: 0.26) 1RM squat, EXP: ↑ 23.7%, NT: ↓ 2% (*p* < 0.001, ES: 1.05) ↑ Box lift (1.5 m), EXP: 10.5%, NT: 3% (*p* < 0.001, ES: 0.27) ↑ Push-up, EXP: 34.5%, NT: 30% (*p* = 0.029, ES: 0.26) Squat jump power, EXP ↑ 7.2%, NT: ↓ 15% (*p* < 0.001, ES: 0.65) ↓ 30-s Wingate mean power, EXP: 4.6%, NT 2% (*p* = 0.282)
Dyrstad et al. (2006)^b^ [51]	107 males, 19.02 ± 0.8 years, ST: 76.8 ± 10.5 kg, NT: 74.2 ± 6.9 kg	Norwegian Army	12 weeks	Cardiorespiratory endurance Upper body endurance	Treadmill stepwise protocol, no change ↑ Sit-ups 55% (*p* < 0.01) ↑ Push-ups 33% (*p* < 0.01) Chin-ups, no change
Williams (2005) [61]	19 males, 18 ± 1 years, 67.9 ± 5.3 kg	British Army	12 weeks	Cardiorespiratory endurance	↑ Multistage fitness test 13.1% (*p* < 0.0005)
Woodhead and Moyinhan (1994) [54]	26 male, 4 female, 24.7 years, weight unspecified	US Navy	14 weeks	Cardiorespiratory endurance Upper-body endurance Upper-body power Lower-body power	↓ 1.5-mile run time 6% (*p* < 0.05) ↑ Sit-ups 18% (*p* < 0.05) Push-ups, no change ↑ Shotput (8 lb) 6.3% (*p* < 0.05) ↑ Vertical jump height 6.3% (*p* < 0.05)
Bell (2000) [26]	509 males, 352 females, 20 years, male: 76.3 ± 12.3 kg, female: 57.8 ± 6.3 kg	US Army	8 weeks	Cardiorespiratory endurance Upper-body endurance	↓ 1 mile run time male: 54%, female: 156% (NS) ↑ Push-up male: 44%, female: 98% (NS) Sit-up male: ↓ 16%, female: ↑ 23% (NS)
Blacker et al. (2009)^b^ [24]	26 males, 28 females, 20.1 ± 3.4 years, 65.3 ± 8.5 kg	British Army	12 weeks	Cardiorespiratory endurance Cardiovascular strain	↓ 2.4 km run time 6% (*p* = 0.001, ES: 0.7) Weeks 1–6: no change, Week 9 ↓ HRR% (*p* < 0.05, ES 1.2)
Laroche et al. (2023) [46]	32 males, 8 females, 24 ± 5 years, weight unspecified	Canadian Military	10 weeks	Cardiorespiratory endurance Sleep duration	↑ *V*O_2_ peak estimation Mean time 5.4 ± 0.4 h per night and decreased to 4.2 ± 0.4 h during field
Richmond et al. (2012)^b^ [50]	30 males, 30 females, male: 18.9 ± 1.6 years, female: 18.6 ± 1.9 years, male: 73.8 ± 12.9 kg, 57.2 ± 6.5 years	British Army	14 weeks	Cardiorespiratory endurance Cardiovascular strain	↑ *V*O_2_ peak estimation 10% (*p* < 0.05) ↑ HRR% male: 32%, female: 31% (*p* = 0.19)
Vogel et al. (1978) [62]	254 males, 19.5 ± 2.4 years, 67.9 ± 9.5 kg	British Army	3 months	Cardiorespiratory endurance Upper-body strength	↑ *V*O_2_ peak estimation 8.9% (*p* < 0.001) Isometric grip strength, no change
Piirainen et al. (2019) [30]	24 males, 18–21 years, NOR: 83.0 ± 21.4 kg, OR: 79.2 ± 19.0 kg	Finnish Military	8 weeks	Upper-body strength Lower-body strength H-reflex and V-wave Neuromuscular function Muscle contraction	Isometric elbow flexion, NOR: ↑ 18% (*p* < 0.01), OR: no change Isometric elbow extension, no change Isometric knee extension NOR: ↑ 19% (*p* < 0.05), OR: no change, H-reflex and V-wave, no change Single twitch (contraction time) NOR: no change, OR: ↑ (*p* < 0.01)
Schram et al. (2024)^a^ [58]	565 males, 364 females, 18–22 years, male: 72.9 ± 12.2 kg, female: 61.6 ± 7.4 kg	US Marines (Navy)	N/A	HR average HR max	↑ to both HR max/average in females compared to males during hikes with 4, 5 and 6 (*p* < 0.001). Intervention featured 6 hikes with ascending loads (1&2: 10 kg, 3&4: 15 kg, 5&6: 20 kg)
Fallowfield et al. (2012)^a^ [73]	12 males, 22 ± 3 years, 80.7 ± 6.8 kg	Royal British Marines (Navy)	N/A	Lower body power	↓ CMJ jump height 8.9% (*p* < 0.001) ↓ CMJ power 5% (*p* < 0.001) (acute change following loaded march)

*CMJ* countermovement jump, *ET* endurance-based intervention, *EXP* experimental group, *HR* heart rate, *HRR%* percentage of heart rate reserve, *JE* Army Junior Entry, *OR* overreached, *NOR* non-overreached, *NS* no statistics, *NT* normal training/control, *RAF* Royal Air Force, *SE* Army senior entry, *ST* strength-based intervention, *TRIMP* training impulse, *1RM* one-repetition maximum

^a^Acute study only

^b^Combined acute and longitudinal study

**Table 2:** Acute studies included from non-recruit populations (*n* = 5)

Study	Subjects (sex, mean age ± SD, weight ± SD)	Branch	Intervention	Outcomes
Armstrong et al. (2023) [2]	12 males, 10 females, male: 23.6 ± 3.6 years, female: 22.8 ± 3.7 years, male: 77.9 ± 8.4 kg, female: 71.4 ± 9.1 kg	British Army	4 × 3-h marches at varying loads (21, 26, 33, 43 kg). Total distance covered: 12.25 km at 4.9 km/h). Followed by cognitive tests (go/no go, n-back, reaction)	↑ *V*O_2_ uptake with load (*p* < 0.01) ↑ work rate in females (*p* < 0.005) ↓ accuracy during n-back and visual go/no go tests
Giles et al. (2019) [23]	31 males, 24.4 ± 4.1 years, weight unspecified	US Army	4-h operational scenario with 2 × 1 h foot marches with 8.8, 47.2 and 50.7 kg across 3 days following by cognitive tests (go/no-go)	↓ Hit rate (*p* < 0.025) and sensitivity (*p* < 0.001) RPE (Borg scale) ↑ with load and time (*p* < 0.001) %HRR ↑ with load (*p* < 0.001), higher values associated with reduced hit rates (*p* = 0.039)
Grenier et al. (2012) [65]	10 males, 38.96 ± 8.9 years, 82.9 ± 9.3 kg	Army (mixed nation, retired)	21 h simulated military mission (27 kg load for battles, 43 kg during marches)	↓ Isometric knee extension, 10.2% (*p* < 0.01) Isometric plantar flexion, no change Twitch interpolation (knee extensor and ankle plantar flexors), origin of fatigue was peripheral for both ↑ Perceived fatigue, 91.6% (*p* < 0.01)
Scales et al. (2021) [60]	21 males, 5 females, NT: 21 ± 5, EXP: 22 ± 6 years, NT: 73.8 ± 34.8, EXP: 78.3 ± 36.8 kg	Civilian	Loaded (32 kg) and unloaded groups marched on a treadmill for 2 h on consecutive days	Isokinetic knee extension, EXP: ↓ on both days (*p* < 0.05). CON: no change *V*O_2_ uptake, EXP: ↑ on day 2, CON: no change
Looney et al. (2018) [59]	8 males, 1 female, 21 ± 3 years, 83.4 ± 12.9 kg	US Army	2 × laps around 2.5-km course, 5 min rest between (repeated with 30% BM (fighting) and 45% BM (approach) loads)	↑ HR in lap 2 for fighting (*p* = 0.01) and approach loads (*p* < 0.01) Respiration rate, fighting load no change, approach load ↑ (*p* < 0.01) ↑ %HRR in lap 2 for both loads (*p* < 0.05) ↑ TRIMP in lap 2 for fighting (*p* = 0.01) and approach loads (*p* < 0.01) Time to completion, no change

*CON* control, *EXP* experimental group, *HR* heart rate, *%HRR* percentage of heart rate reserve, *NT* normal training/control, *RPE* rating of perceived exertion, *TRIMP* training impulse

### Initial Military Training Structure

Much of the identified literature did not clearly detail the structure or training regime. Of the available information, the duration of each course varied substantially (5–18 weeks) and included both voluntary and conscription-based (compulsory military service) recruitment. Two key phases were commonly reported, with the initial phase spent in team building and on basic skill work such as marksmanship, marching, drill, tactics and physical training [15, 24, 44–46]. The latter phase included field/scenario-based training [45–47], following a progressive model with increased exposure to physical stress as the course progresses [24]. Daily energy expenditure has been estimated at around 18,000 kJ [45, 48], comparable to that of elite athletes [16]. Of the research that reported sleep [21, 42, 47, 48], recruits slept between 5.4 h and 6.5 h per night. This was, however, variable across studies and within phases of IMT, with decreases in sleep duration as low as 4.2 ± 0.4 h [46] and subjective quality noted in field environments [21].

One study of Finnish conscripts [49] noted that approximately 40% of the time spent training involved a physical element, with around 13% dedicated to improving physiological and neuromuscular qualities. Another study reported over 2 h of physical activity being conducted daily (males: 2.3 ± 0.2 h, females: 2.2 ± 0.2 h) [50]. Research on Australian Army recruits suggests that < 10% time is spent on specific physical training sessions, equating to around 250 min per week [45]. This is similar to the US, British and Singaporean armies’ average of 227, 284 and 240 min per week, respectively [48]. This contrasts with Dyrstad et al. [51], who state that the minimum standard for specific physical training during IMT in the Norwegian military is only 120 min per week (with only 12% of the cohort meeting the minimum standard). While these variations may reflect differences in what is classified as targeted conditioning for specific physical qualities, it nonetheless highlights the large differences possible between organisations. Time spent on structured physical training appears to be largely divided up between forms of cardiovascular and strength training, utilising running, strength, circuit training, sport, swimming and obstacle courses [45, 48, 52]. These training sessions run for approximately 1 h, occur multiple times a week [35, 51, 52] and usually lack individualisation [53]. Most courses adopt traditional training methodology and focus on prolonged low-to-moderate intensity endurance-based training. Few explicitly identified alternate methods, such as properly structured strength training, which could be argued as beneficial for adaptation of key physical qualities for potential soldiers [34, 37, 38, 40].

Outside of structured training sessions, recruits’ time was separated by specific skills such as weapons training, drill, field craft and classroom sessions and other physical activity [36, 40, 48]. The remaining physical activity was spread across a range of different activities, such as load carriage, which was among the most reported and investigated activities identified in the literature search [35, 39, 41, 48, 53]. In addition, one study on Swiss recruits noted that the average recruit was covering 14 km per day on foot [48], with another study noting that most distance covered on foot was conducted at ‘double time’, referring to running [54]. While physical activity varies between organisations, generally, the total duration appears very high among recruits.

### Physiological and Neuromuscular Effects of Initial Military Training

*Cardiorespiratory system* Cardiorespiratory endurance is the heart and lungs’ ability to deliver oxygen to muscles [55]. It is a critical factor for sustaining physical output over time [56, 57]. Enhanced endurance improves oxygen uptake and exercise economy, making it perhaps the most vital physical quality for tactical personnel, particularly during prolonged load carriage [50].

*Acute Effects* Acute cardiorespiratory responses were discussed in four studies [24, 50, 51, 58]. Three studies investigated the responses of heart rate during fitness tests (multi-stage fitness test, 2.4-km run and during *V*O_2_ peak testing) [24, 50, 51] and one during an intervention involving six hikes with progressively increasing loads (total duration 225 min) [58], which was the only study to investigate acute aerobic responses to a military-specific task. In another study, the daily average for percentage heart rate reserve (HRR), which is used to measure cardiovascular strain (higher percentages represent greater strain), was higher in the initial stages of IMT, but there were no changes between weeks 1 and 6 (31% ± 5% versus 31% ± 5% HRR, *p* = 0.246, d = < 0.1) but was significantly lower in week 9 (29% ± 5% HRR, *p* < 0.05, d = 1.2) [24]. Accordingly, Richmond et al. [50] observed that week 1 had the highest %HRR for both male and female British recruits, but throughout IMT, there were no daily differences between sexes despite lower comparative fitness levels recorded in the female group. However, there were significant differences detected within individual weeks for both sexes. By contrast, another study found that HRR% was significantly lower in British male than female recruits (24% ± 2% versus 32% ± 2% HRR, *p* < 0.001, d = 4.0) [24], likely due to lower cardiorespiratory endurance rather than sex alone (females had significantly lower levels compared with males).

During loaded and unloaded marching over varying distances (3–12.25 km) and durations (single short bouts to multiple-day repeated bouts), increases in intensity and or load increased cardiovascular strain [23, 51, 58, 59] and *V*O_2_ consumption [2, 25, 60] in both male and female recruits. Overall, females worked at 33% greater percentage of their maximal oxygen consumption (%*V*O_2_ max) than males under the same conditions [25]. In addition, as the required load carriage was increased, so too were the physical demands placed on personnel [2, 58, 59], showing that an increase in load equivalent to 15% body mass increases %HRR by around 10% [59]. Of these studies, Armstrong et al. [2] and Looney et al. [38] investigated the effects of repeated bouts of load carriage, with both studies finding significant increases in measures of physical strain in both sexes. Armstrong et al. [1] found an increase in 10% HRR compared with the first march in males and 19% in females. While Looney et al. [59] tested eight males and one female in three different loading conditions: unloaded, fighting load (30% body mass, BM) and approach load (45% BM). Both fighting and approach loads showed significant increases in HR, %HRR and training impulse in the second bout of marches, while for respiration rate (RR), only the approach load increased significantly (all *p* ≤ 0.01).

*Chronic Effects* The chronic effects of IMT on the cardiorespiratory system were included in 21 studies [24, 26, 32, 34–41, 44–46, 50–54, 61, 62] assessed via a range of different methods over 5–16 weeks; for example, multistage fitness tests [37–39, 45, 50, 53, 61], runs for time at varying distances (1.6, 2, 2.4 and 3 km) [24, 26, 36, 41, 44, 50, 54], loaded runs with varying loads for 3.2 km (18, 22 and 32 kg) [32, 35, 37, 38], shuttle runs [35, 36], Coopers 12-min Test [39, 52], and treadmill and bicycle *V*O_2_ analysis tests, both using estimation and gas analysis [32, 34, 35, 40, 46, 62]. Among these, evidence suggests that IMT increases cardiorespiratory endurance, although the magnitude varies substantially (− 6% to 61%), with the average effect appearing positive. These findings align with a review of 29 studies on military recruits across the UK, USA, Australia, Canada, South Africa and Germany [3] which reported improvements in all studies across male and female recruits, with increases in *V*O_2_ max of 7.4% in males and 4.0% in females. Of the 21 studies included in the current review, all generally observed positive improvements. Nonetheless, two studies during a 12-week block of IMT included a stepwise treadmill protocol (*V*O_2_ peak via gas analysis), which found no significant change [51], and a 2.4-km time trial, finding a 6% decrease (*p* < 0.001) [36]. This broad variation is likely due to differences in training focus, progression and IMT duration, details of which are not always clearly reported, as described earlier.

Most improvements to cardiorespiratory endurance appear to occur within the first 8–10 weeks of IMT, and in those with the lowest entry fitness levels [34, 51], which might suggest that IMT initially is sufficient to evoke improvement in cardiorespiratory endurance, after which it is then potentially obscured by a lack of appropriate periodisation. Consequently, because studies on shorter-duration programmes have not reported intermediary data points, it remains unclear precisely how these shorter timelines alter the trajectory of such adaptations. In Australian, British and Finnish Army and Air Force recruits, mean pre- and post-IMT differences in aerobic performance have largely been driven by individuals with the lowest levels of initial fitness [34, 40, 41, 53], while those with a *V*O_2_ peak above 55.5 ml/kg/min made no significant progress [40]. This is supported by Dyrstad et al. [51], who found that a medium initial VO_2_ peak (54.6 ml/kg/min) exhibited no change, and a high initial *V*O_2_ (59.7 ml/kg/min) experienced a 1.1% decrease (albeit not statistically significantly, *p* = 0.07). These findings suggest that IMT is broadly insufficient in improving cardiorespiratory endurance in those with exceptional fitness levels, and that early increases may simply be attributed to lower starting fitness and may not continue to be developed during the later stages of IMT.

Furthermore, in a study of British recruits, the magnitude of improvements in recruits tended to vary by sex. However, the primary explanation appears to be that males expressed higher fitness levels at the start of the course [24], in line with previous findings [40, 53]. This is further supported by the findings that, while female recruits have 2.1 times greater relative injury risk compared with male recruits, this difference becomes negligible when adjusted for fitness [26]. These results suggest a relationship between cardiorespiratory endurance and injury during IMT, which suggests that those working at a greater relative level of strain (notably females) are at higher risk [24].

*Summary* The literature suggests that IMT acutely increases cardiovascular strain while chronically improving cardiorespiratory endurance. These improvements appear to be largely attributed to lower entry-level fitness, and progress seems to stall in the latter stages of IMT, with those entering with a high level of cardiorespiratory endurance showing trivial progress throughout the duration of IMT. Although the reported studies hypothesise different reasons for these effects, this is likely a result of compounding variables such as chronic poor sleep quality, peripheral fatigue due to insufficient recovery and a lack of individualisation of training. Nonetheless, variation in testing methodology, time between pre/post measures, and differences in underlying characteristics of IMT complicate further synthesis.

**Maximal strength** Muscular strength is the maximum force that muscles exert on their environment [63]. Strength training (ST) can reduce injury risk by up to 66% [64] and significantly improve performance in demanding tasks such as load carriage, lifting and casualty evacuation [5, 8]. These benefits are further amplified when strength work is combined with aerobic training.

*Acute Effects* One study [32] investigated the acute effects of military IMT on maximal strength. In this study, male Finnish Army conscripts, divided into groups prioritising strength, endurance or normal training, completed a timed 3-km loaded run with 14.2 kg of additional load. Interestingly, all groups experienced a significant decrease in knee extensor strength by approximately 10% (*p* = 0.01–0.05) immediately following the activity. Other studies conducted in military populations outside of IMT reported similar lower-limb strength output decreases of around 10% (isometric knee extension and plantar flexion) immediately following load carriage [60, 65], which can persist up to 96 h [66]. Only one paper was able to infer that this was isolated to peripheral fatigue by using twitch interpolation on the knee extensors and plantar flexors; however, it featured a non-recruit population [65]. Logically, these effects tend to scale with relative load increase, placing greater stress on the musculature [25], which is notable for those of smaller stature or strength in conditions of absolute loading. While there is scarce literature specific to IMT, it seems that loaded tasks impair maximal muscular strength in the short term.

*Chronic Effects* Ten studies investigated the chronic effects of IMT on maximal strength measured over 8–16 weeks [30, 32, 34, 35, 37, 38, 40–42, 62]. Of these, one paper focused solely on upper-body effects [62], five on lower-body effects [32, 37, 40–42] and four on both upper- and lower-body effects [30, 34, 35, 38].

Upper-body strength was assessed using a 1RM bench press over 8 and 12 weeks [35, 38], an isometric bench press over 16 weeks [34], isometric elbow flexion and extension over 8 weeks [30] and an isometric grip strength test over 3 months [62]. Four of these studies showed statistically significant improvements in bench press and isometric elbow flexion [30, 34, 35, 38], while the elbow extension and grip strength showed no change [30, 62]. A recent review of recruits [3] reported median improvements of 8.5%, with larger improvements typically observed in females (6.9% versus 13.0%). These findings align with those found during the literature search.

Lower-body strength was assessed using a variety of different methods, including a 1RM squat over 8 and 12 weeks [35, 38], 1RM box lift (1.5 m) over 12 weeks [37, 38], isometric mid-thigh pull (IMTP) over 12 and 13 weeks [41, 42] and an isometric knee extension over 8 weeks [30]. Within these studies, four found overall improvements [30, 35, 37, 41], one found no change [42] and one was relatively inconclusive, where the 1RM box lift had improved by 3%, and 1RM squat decreased 2% (percentages calculated from data) [38]. In line with the effects of cardiorespiratory endurance, the effects of IMT on lower-body strength also appear more pronounced in those with lower initial strength; notably, where recruits in the lowest quartile of strength saw a 24.4% increase in IMTP force (*p* < 0.001), whereas the highest quartile showed no change (− 2.8%, *p* = 0.202) [41]. While these results seem to show generally positive improvements overall, the identified literature itself is inconclusive and features significant variations in testing methodology and outcome. Notably, a previous systematic review of pre and post changes to physical performance in military training by Varley-Campbell et al. [3] observed an overall significant improvement in lower-body strength of 8.9%. The majority of the identified literature in this review observed positive changes in 14 total groups, while 5 returned no significant difference. This seems to align with the literature identified in the current review, where improvements to lower-body strength are observed in four studies, while two found no difference.

When looking more specifically at studies which tested strength during several timepoints in IMT, we see similar effects as with the cardiorespiratory adaptations, where most improvements are made within the initial periods of IMT where isometric elbow and knee extension strength increased by 3.8% and 8.1% (*p* < 0.001) in the first 8 weeks and no additional improvements were made in the remaining 8 weeks [34]. The authors attributed these inconsistent results to an interference effect or lack of progressive stimulus, but given that this effect appears to occur across other physical qualities as well, accumulated general fatigue may be another explanation. In addition, it appears that most positive adaptations take place within those with the lowest levels of entry strength [41]. Only one of the studies sought to investigate sex-based differences [42], finding that males had greater IMTP peak force (*p* < 0.001), albeit no clear difference when normalised to body mass (*p* = 0.09).

Large variances exist in the physical training regimen, but most tended to adhere to a traditional, moderate-to-low endurance-based load, which is suboptimal for maximal strength development. Studies that featured an experimental design comparing a control group (NT), which performed an unmodified IMT, with a group that conducted an alternate strength training (ST) programme with better control over volume and load, also found more favourable results in the ST groups [38, 40]. Both studies featured a design where the ST group performed strength and high-intensity interval sessions. The study by Santtila et al. [40] found an increase of 9.1% (*p* < 0.05) during isometric leg extension and elbow extension, whereas there was no change observed in the NT group over the course of IMT (5.2%, *p* = 0.45). This is supported by Burley et al. [38], who found that, compared with the NT group, the ST group experienced small improvements in load carriage, 1RM bench press and 1RM box lift, with large improvements in 1RM squat. By contrast, a study that compared standard IMT with a circuit training protocol found no significant difference in strength for 1RM bench press or squat [35]. Utilising a circuit style protocol with very short rest periods has been shown to produce suboptimal strength improvements [67, 68]. These findings suggest that a more targeted approach to training results in less fatigue and offers more favourable results.

*Summary* IMT (and associated military tasks) appear to acutely suppress strength and scale to relevant activities. Chronic effects overall seem to elicit improvements in strength, although results are inconsistent and heterogeneity is present amongst testing methodologies. This may, in part, be due to different organisational practices and training methodology, and varying testing methodology and timeframes, as described earlier. To support this hypothesis, most observed improvements were made early in the IMT and largely within those in the lowest levels of strength, suggesting that fatigue might play a role in these effects. Furthermore, intervention studies featuring groups with more strength-based volume, better control over load and rest periods tended to see better improvements to maximal strength. To enhance and optimise maximal strength adaptations, military practitioners might consider a more periodised approach to their training, adjusting endurance loading to allow better strength adaptations.

*Muscular endurance* Neuromuscular endurance is a critical component of military readiness, allowing recruits to sustain maximal effort [69] and perform repeated operational tasks such as load carriage. Despite its importance in IMT, the multifaceted nature of force and velocity makes standardised assessment difficult.

*Acute Effects* There were no studies identified that investigated the acute effects of IMT on muscular endurance. However, the impacts of difficult activities are reasonably well understood in human physiology and may persist for up to 96 h in soldiers [66]. The negative effects of IMT observed in strength likely transfer to strength endurance. For example, one study [32] identified that knee extensor strength declined 10% in male Finnish Army conscripts following a 3-km loaded run (14.2 kg). Byrne et al. [70] reviewed evidence showing that repeated submaximal eccentric exercise causes greater muscle function impairment compared with concentric exercise, suggesting that increased muscle damage further diminishes muscular endurance and stretch–shortening cycle efficiency during repetitive activities such as running. Given the intense repeated physical demands of IMT, it is likely that these effects would also be observed in recruits during IMT.

*Chronic Effects* Eight studies have looked at the chronic effects of IMT on muscular endurance measured over 5–14 weeks [26, 35–38, 44, 51, 54], typically determined using maximum repetitions in sit-ups, push-ups and chin-ups. Given the endurance-based nature of IMT [15], it may come as no surprise that all eight studies showed significant improvements in muscular endurance between 5 and 156% over the duration of the course (calculated manually across studies). However, all endurance-based tests used an upper-body focus, and no lower-body measures were used. In the absence of sufficient literature, some other tests that were deemed to be more aerobic in nature may be used to strengthen this section. The tests, including a 400-m loaded run, shuttle runs, an obstacle course and a casualty rescue [35, 36], showed performance improvements between 6 and 36% across all measures. Averages calculated manually across studies from the provided data.

The chronic improvements in muscular endurance resulting from IMT seem to be greater in female recruits, with Bell [26] showing that females improved more than males in the push-up (156% versus 54%) and sit-up (98% versus 44%, respectively) over 8 weeks. These findings align with systematic research, which shows that muscular endurance is significantly improved for both men and women during IMT [3, 71], averaging 52% for push-ups and 47% for sit-ups, with females showing the greatest improvements in both push-ups (71% versus 50%) and sit-ups (53% versus 36%) [18]. Furthermore, the testing methodology identified was primarily upper limb dominant, with just a single paper identified in the Varley-Campbell et al. [3] systematic review investigating lower limb endurance using a leg press until failure, finding no significant differences between male and female recruits. Ultimately, larger improvements were observed in females’ upper-body strength endurance, likely attributed to lower initial strength levels, which are markedly lower among female compared with male active populations [3], but lower-body endurance requires more research.

*Summary* While acute responses to IMT have not been documented in recruit studies, broader research suggests that neuromuscular endurance is likely impaired following strenuous physical activity. This impairment tends to increase with greater muscle damage, especially from eccentric loading, such as that experienced during cross-terrain marching, where downhill sections impose high eccentric demands. Overall, IMT has resulted in a significant improvement in muscular endurance in both male and female recruits, but there were no studies identified in recruits that measured lower-body endurance objectively and found no significant differences. Systematic research in recruits supports these results for upper-body endurance, noting that significant improvements were seen over the duration of the course, with females making the largest changes. Where testing methodologies were more closely aligned and comparable than other qualities assessed, timeframes still varied significantly. Further research is required with lower-body assessments to draw valid conclusions, with only a single study identified in systematic research, which found no change between sexes.

*Muscular power* Neuromuscular power is the ability to produce force rapidly and is essential for military performance [6]. High power levels enhance explosive efforts and agility [72], enabling personnel to manoeuvre quickly, navigate obstacles and perform forceful tasks such as throwing or seeking cover.

*Acute Effects* In the only study that we found that examined the acute effects of IMT on neuromuscular power, Fallowfield et al. [73] investigated the acute effects on an all-male sample of a 19.3-km loaded march taking 270 min to complete while carrying 31 kg of additional load. Lower-body power was measured with a countermovement jump (CMJ) on a jump mat, which is a commonly used assessment to measure lower-body power [74]. Jump height and power (calculated using an equation) were recorded. They found that, immediately following the loaded march, there was a decrease of 8% ± 9% in jump height, and power decreased 5% ± 5% (*p* < 0.001). These findings align with previous research showing decreases in neuromuscular function following endurance activities [75]. Similarly, a study on a civilian male cohort performing loaded carries reported a 9% ± 6% decrease in CMJ jump height [76], findings that closely mirror those in a military sample [73]. Laboratory studies have shown that these impairments might last up to 48 h post activity [75], while a military-specific systematic review reported that power may take as long as 96 h to recover fully following periods of field training and casualty evacuation exercises [66], highlighting the significant fatigue placed on military personnel. None of these studies featured female subjects, and as such, further research is required to understand the interaction between sex and acute changes to neuromuscular power.

*Chronic Effects* The chronic effects of IMT on neuromuscular power were investigated in six studies, measured over 8–14 weeks [35, 38, 39, 41, 42, 54]. Two investigated upper-body power via a medicine ball throw over 13 weeks [41] and shot-put throw over 14 weeks [54], while 5 of these studies investigated lower-body power via vertical and horizontal jumps, or a Wingate 30 s test over 8–14 weeks [35, 38, 39, 42, 54]. Again, there are large variations between test timing, methods and population.

Both upper-body studies utilised a projectile throw/push-based test. Upper-body power is important for personnel as it allows for quick movement from prone positions, which are often utilised during combat scenarios, and may assist with the movement of objects. Woodhead and Moyinhan [54] found a significant 6.3% increase (*p* < 0.05) while Rue et al. [41] investigated the upper-body power in three different branches of the military with improvements of 6.8% (*p* < 0.001) and 2.3% (*p* = 0.040) for British Army Junior Entry and Army Senior Entry, respectively, while the Air Force decreased 1.4% (*p* = 0.002) performance. These groups were all ranked into quartiles based on their throwing distance. The increases in the Army courses were made up by those who were in the lowest half of upper-body power within the cohort of both Army Junior and Senior entry. Only the lowest quartile made any improvement in the Air Force group, which suggests that the training stimulus may not be sufficient to enhance upper-body power in recruits with reasonably developed upper-body power. It is unclear exactly why these adaptations occurred; neither study made specific mention of why they believe this happened, but these can probably be attributed to general exposure to structured training, which seems likely due to most improvements being observed in recruits with lower levels of power on entry.

The results for the chronic effects of IMT on lower-body power were mixed. Three studies reported significant decreases averaging 8.9% (− 2% to − 15%) [38, 39, 42], while two others observed significant increases averaging 3% (1% to 6%) [35, 54], with means calculated manually from the provided data within each paper. Interestingly, both papers showing improvements to lower-body power used manually measured vertical and horizontal jumps, with only small increases detected of 1%, 3% and 6% for each of these conducted tests [35, 54]. The remaining three papers, which observed decreases in power using more advanced technology, such as force plates, jump mats and a cycle ergometer, all found decreases in power (2–15%) [38, 39, 42]. Only one of these explored the interaction of sex [42], finding that females’ peak power and relative peak power from a CMJ measured on force plates had decreased significantly less than males’ (*p* < 0.001). Further research is needed to draw valid conclusions about the chronic effects of IMT on lower-body neuromuscular power.

*Summary* In line with current understanding in athletic populations, muscular power is reduced following strenuous activity. Acute responses to physical activity in recruits showed decreases in power, with one study suggesting that males suffered greater reductions over the course of IMT than females. While research was limited and, again, large variances in testing methodologies and timeframes exist, these findings seem to align with a greater body of research in wider military and civilian populations. However, the chronic effects of IMT on neuromuscular power are currently inconclusive. Limited research on upper-body power showed consistent increases across both studies. By contrast, findings on lower-body power were more variable.

## Limitations and Future Directions

The heterogeneity of the included studies presents a challenge to coherently summarising the effects of IMT. As described, studies include IMT instances that span countries, timeframes and different measurement techniques, which render synthesis challenging. Specifically, the difference between service types, such as conscript versus volunteer populations, introduces significant variance in motivation and pre-enlistment conditioning. Furthermore, baseline fitness levels varied widely among cohorts, complicating the ability to isolate the specific impact of initial military training (IMT). This is exacerbated by the inability to control for variables known to influence performance, such as sleep deprivation, nutritional intake and psychological stress, which are common to the military environment. The training itself presented further inconsistencies and lacked clarity, likely in part due to security risks with sharing a detailed training regimen. In many cases, mass training environments limit the feasibility of individualised training methodologies, and frequent shifts in training focus often occur throughout the course. In addition, the varying lengths of training courses and the specific requirements of different military branches (e.g. Army versus Air Force versus Navy) limit the generalisability of the results. Finally, the data may be affected by the nature of the assessments themselves; testing may not have been conducted maximally due to recruit fatigue or a lack of standardised motivation, potentially resulting in underestimations of true neuromuscular capacity and obfuscation of true effects.

Future research should prioritise the establishment of standardised physical assessment protocols across military cohorts, potentially leveraging more insightful measurement approaches and technologies. Large-scale, longitudinal studies with similar data collection methods are essential to improve comparability and to develop a more cohesive understanding of how specific training modalities influence long-term health and readiness.

## Conclusion and Research Suggestions

This review examines how initial military training (IMT) impacts physiological and neuromuscular performance. Although specific data on recruits are sparse, related research indicates that IMT tasks likely reduce acute neuromuscular and cognitive output. This decline is likely a necessary byproduct of the preparatory stress intended to challenge recruits, rather than solely to stimulate positive long-term physiological adaptations.

The chronic IMT adaptations generally improve cardiorespiratory endurance, strength and upper-body muscular endurance; however, data on lower-body endurance and muscular power are underreported and remain inconclusive. Notably, improvements were predominantly observed in groups with the lowest initial fitness levels, whereas the fittest groups showed minimal or no gains. Furthermore, these adaptations primarily occur during the initial phases of IMT, with progress stalling as training continues, resulting in little to no additional improvement during the latter stages of the course. This plateau, likely driven by a lack of appropriate periodisation, increases injury risk and compromises safety. Research suggests that reducing endurance-based loads offers more favourable outcomes for strength and power. Consequently, tracking fatigue proxies is essential to balance stressors with recovery, ultimately enhancing performance and reducing the high injury rates common in IMT [10, 26, 77]. It is important, however, to reiterate the significant heterogeneity observed across the included studies, which makes a concise synthesis difficult and calls for more specific research.

These physiological responses are further nuanced by sex-based differences in baseline conditioning and training strain. For instance, females typically enter IMT with lower initial fitness levels and consequently exhibit greater cardiovascular strain during load carriage. While both sexes show improvements in muscular endurance, these effects were more pronounced in females. Males maintain higher absolute isometric strength. However, the impact of cumulative fatigue also appears to diverge; limited data suggest that males may experience a greater reduction in jump power than females over the duration of IMT. These sex-specific variations highlight the importance of a degree of individualised monitoring to mitigate the high injury rates associated with the cumulative stress of training.
